# Prevalence and correlates of overweight and obesity among under-five children in Egypt

**DOI:** 10.3389/fpubh.2022.1067522

**Published:** 2022-12-14

**Authors:** Emmanuel Osei Bonsu, Isaac Yeboah Addo

**Affiliations:** ^1^Department of Epidemiology and Biostatistics, Kwame Nkrumah University of Science and Technology, Kumasi, Ghana; ^2^Centre for Social Research in Health, University of New South Wales, Sydney, NSW, Australia

**Keywords:** child overnutrition in Egypt, overweight and obesity, under-five children, risk factors, excess body weight, overnutrition, Demographic and Health Survey

## Abstract

**Background:**

Evidence suggests that Egypt, a country in North Africa, has a significant number of children at serious risk of excess body weight. Yet, there is a dearth of studies on overweight and obesity among children under 5 years in the country. This study examined the prevalence and correlates of overweight and obesity among under-five children in Egypt.

**Methods:**

Data were retrieved from the latest (2008 and 2014) Egypt Demographic and Health Surveys (EDHS). A total of 42,568 children under 5 years were included. The prevalence of overweight and obesity was described using proportions whereas the factors associated with the prevalence were examined using logistic regression.

**Results:**

Of the 42,568 children under 5 years, about one in every six (17%) were overweight or obese. Children aged 19–37 months, those with birth weights >4 kg, those given large portions of protein foods (eggs and meat), and those whose mothers were in the rich wealth quintile had significant risks of overweight or obesity.

**Conclusion:**

Overweight and obesity are highly prevalent among children under 5 years in Egypt. Interventions developed to address these two overnutrition indicators in Egypt need to consider variations in risk factors across age, birth weight, food types and portions, and maternal wealth status.

## Introduction

Overweight and obesity defined as an abnormal or excessive build-up of body fats that might harm one's health (1), are emerging public health concerns in Africa (2). The impact of overweight and obesity is even more significant when it occurs in children as it puts them at increased risk of both early and long-term breathing problems, musculoskeletal diseases, hypertension, diabetes, stroke, insulin resistance, some cancers, and adult obesity (3, 4). Also, obesity in childhood is linked to a higher risk of mental health issues, impairment, and even death (5).

Globally, there are about 41 million children under 5 years reported to be either overweight or obese as of 2018 (6). A growing body of evidence shows that the current majority of obese or overweight children are in developing countries, where the rate of increase is more than 30 percent higher than in industrialized countries (7). Overweight and obesity in children are on the rise, largely, due to significant changes in dietary and physical activity behavior (8). Thus, technological advancement has made it easier to perform daily physical activities; hence increasing sedentary lifestyles and reducing physical activities (9). In recent years, more children are also spending more time indoors and are likely to spend much time watching television or playing video games, which makes them less active and gain weight as a result (10). Moreover, dietary patterns have changed from traditional healthy diets largely composed of fruits and vegetables to energy-dense foods which are high in calories, fats and sugars (11). The World Health Organization recommends that to avoid the risk of excess body weight, infants should be breastfed exclusively during the first 6 months of their lives, should be breastfed continuously until at least 2 years of age, breast milk should be complemented with a variety of nutritious foods, salt or sugar should not be added to foods, food portions should contain at least 400 g or five portions of fruits and vegetables per day, and fat intake should be <30% of total energy intake (12).

Evidence shows that genetic or family characteristics are also factors associated with risk of overweight and obesity (13). Research indicates a possibility of a person inheriting about 40% of body type from parents (14). Additionally, emerging evidence suggests that socio-demographic factors, such as parental wealth status and place of residence have a significant impact on the likelihood of childhood overweight or obesity (15). Importantly, overweight or obesity resulting from socio-demographic factors are known to be modifiable compared to genetic factors (13). This idea of modifiable and non-modifiable risk factors associated with overweight and obesity is critical to developing appropriate interventions for reducing childhood overnutrition.

One of the regions in the world facing a high risk of overnutrition is North Africa. Studies show a rising trend in childhood overweight and obesity in North Africa, especially, in Egypt, with the prevalence higher than most countries in the Middle East and Sub-Saharan Africa (15, 16). A study among school-going children in Sohag, Egypt found that 17% of the participants were overweight and 15% were obese (3). Behera, a suburb of Egypt found as high as 18% of overweight and obesity among primary school children in 2019 (17). Although several community-based evidence shows a high prevalence of overweight and obesity among children under-5 years in Egypt, childhood overnutrition is under-studied in Egypt with most studies focusing on adults and adolescents (3, 17). In view of this significant gap, we examined the prevalence and correlates of overweight and obesity in Egypt. Findings from this study are critical to developing tailored interventions aimed at addressing overnutrition in the country and even beyond.

## Methods

### Overview

Since 1980, several surveys have been carried out in Egypt to obtain data from the community on the current health situation including a series of Demographic and Health Surveys (DHS) of which the 2014 Egypt Demographic and Health Survey (EDHS) is the most recent (18). The 2014 EDHS is of special importance as it is the latest and first national health survey since 2008. The initial results of the 2014 EDHS show that key maternal and child health indicators, including antenatal care coverage and medical assistance at delivery, have improved. However, the survey also documents several critical challenges, particularly relating to fertility and family planning (18). The findings of the 2014 EDHS together with the service-based data are very important for measuring the achievements of health and population programs. The 2014 EDHS was conducted under the jurisdiction of the Ministry of Health and Population (18). International classification of functioning, disability and Health (ICF) provided technical support for the survey through the DHS program. The DHS Program is sponsored by the United States Agency for International Development (USAID) to assist countries worldwide to obtain information on key population and health indicators (18). USAID/Cairo also provided funding to support the implementation of the survey. UNICEF and UNFPA also contributed funding to the survey. The 2014 EDHS survey design has two components; a survey of ever-married women aged 15–49 years and a special Health Issues survey to obtain updated information on other critical health problems facing Egypt (18). The data are publicly available at http://measuredhs.org. Details on the approach used in gathering the data including the sampling methods can be found in the EDHS reports (18, 19).

### Data

This study was based on the latest [2014 (EGKR61DT.ZIP) and 2008 (EGKR61DT.ZIP)] children's data drawn from Egypt's Demographic and Health Surveys (EDHS). The EDHS children's data contained information on children's nutrition and women aged 15–49 years. Approximately, 42,589 children under five were sampled to partake in the study (18, 19).

### Variable description

A total of 18 variables were included in the study, and these variables were categorized into three: (i) Child variables which included age (0–18 months = infant, 19–37 months = toddlers, 38–59 months = children), sex (males and females), birth weight (<2.5 kg = low birth weight, 2.5–4.0 kg = Normal weight, 4.1 and above = overweight), place of residence, access to a bicycle, access to vehicle, child given carbohydrate foods, child given protein foods, child given fatty foods and child given fruits, (ii) Maternal variables including maternal age (11–19 years = adolescent, 20–28 years = young adult, 29 years and above = adult), educational level, wealth index, maternal BMI (when BMI < 25 kg/m^2^ = Not overweight/obese, when BMI > 25 kg/m^2^ = Overweight/obese) (20), marital status (married = currently married, widow+divorce+never married = not married), postnatal visit, and current work status), (iii) Husband/partner's educational level (Table 1).

**Table 1:** Variable categorization and description.

**Variables**	**Coding used for the analysis**
**Outcome**	
Overweight/obese	1 = *z*-score ≥ 2
Not overweight/obese	0 = *z*-score < 2
**Independent**	
Age of child	1 = 0–18 months, 2 = 19–37 months, 3 = 38+ months
Sex of child	1 = Male, 2 = Female
Birth weight	1 = < 2.5 kg, 2 = 2.5–4.0 kg, 3 = 4.1+ kg
Access to bicycle	1 = No 2 = Yes
Access to car	1 = No 2 = Yes
Child given carbohydrate food	1 = No 2 = Yes
Child given protein food	0 = No, 1 = Yes
Child given fatty food	0 = No, 1 = Yes
Child given fruit	0 = No, 1 = Yes
Maternal age at first birth	1 = 11–19 years, 2 = 20–28 years, 3 = 29+ years
Education level	0 = No formal education, 1 = Primary level education, 2 = Secondary level education, 3 = Higher level education
Place of residence	1 = Urban, 2 = Rural
Wealth index	1 = Poor, 2 = Middle, 3 = Rich
Mother's BMI	1 = Overweight/obese, 2 = Not overweight/obese
Marital status	1 = Not married, 2 = Married
Antenatal visit during pregnancy	0 = No, 1 = Yes
Postnatal visit	0 = No, 1 = Yes
Father's educational level	0 = No formal education, 1 = Primary level education, 2 = Secondary level education, 3 = Higher level education
Current work status	1= Not working, 2 = Working

Body mass index (BMI) was measured and calculated using the WHO's new standard for child growth (21). The new standard is an international standard for assessing nutritional status, physical growth, and child development from birth to the 5th year. Overnutrition (overweight and obesity) was calculated in standard deviation using the *z*-score ≥ 2. Childhood overweight/obese was defined as *z*-scores ≥ 2. Also, the mother's overweight/obese was considered a BMI > 25 kg/m^2^ (21). To determine the actual child BMI value in the datasets, the measure was divided by 100.

With regards to other covariates, we assessed child food consumption by these questions: “Did you give your child eggs and meat (protein food)?” “Did you give your child any other fruits?” and “Did you give your child oil, fats, and butter products?” The responses to these questions are described in Table 1.

### Data analysis

Descriptive statistics including frequencies and percentages were performed. Aside from the descriptive statistics multivariate analyses (logistic regression) were computed in the final model to observe associations between the independent and dependent variables. Based on recommendations from empirical literature (15), the logistic regression analysis was set at a 95% confidence interval and adjusted for other covariates which included: age of child, sex of child, birthweight, place of residence, access to car, access to bicycle, child given carbohydrate, child given protein food, child given fatty foods, child given fruits, maternal age at first birth, mother's educational level, wealth index, mother's BMI, antenatal visit, postnatal visit, father's educational level, and current work status. Logistic regression was used because the outcome variable was categorized into two, Overweight/obese = 1 and Not overweight/obese = 2. The analysis was performed using Stata/SE 14.

## Results

### Socio-demographic characteristics of participants

A total of 42,568 children under 5 years were included in the study. Of these, there was a similar proportion in terms of age. A little more than one-third (34%) and (33%) were between 0 and 18 months and above 38 months, respectively. With regards to sex, the majority (52%) were males and the remaining 48% were females. Children who were born with low birth weights were ~15% whereas the majority (83%) weighed between 2.5 and 4.0 kg at birth, with only 2% weighing above 4.0 kg. Most (68%) of the children lived in rural areas whereas the remaining 32% were in urban areas. Less than 10% had access to a bicycle while the majority (93%) had access to a car. In terms of food consumption, about 4 percent were commonly given carbohydrate foods whereas more than a quarter (30%) were commonly given protein foods. More than half (56.4%) were commonly consuming fatty foods whereas less than a quarter (8.8%) were commonly consuming fruits (Table 2).

**Table 2:** Participant characteristics.

**Variable**	***N* = 42,568** **(%)**	**Weighted** **% (CI)**
**Child variables**
**Age of child (months)**		
0–18 months	14, 568 (34.25)	34.08 (0.33–0.35)
19–37 months	13,642 (32.01)	32.01 (0.31–0.33)
38+ months	14,358 (33.74)	33.91 (0.33–0.35)
**Sex of child**		
Male	22,057 (51.82)	52.20 (0.51–0.53)
Female	20,511 (48.18)	47.80 (0.47–0.49)
**Birth weight**		
< 2.5 kg	3,326 (14.08)	14.67 (0.14–0.16)
2.5–4.0 kg	19,662 (83.24)	82.90 (0.82–0.84)
>4.0 kg	633 (2.68)	2.42 (0.02–0.03)
**Place of residence**		
Urban	16,771 (39.40)	32.47 (0.31–0.34)
Rural	25,797 (60.60)	67.52 (0.66–0.69)
**Access to bicycle**		
No	37,299 (92.72)	92.84 (0.92–0.93)
Yes	2,927 (7.28)	7.16 (0.07–0.08)
**Access to car**		
No	36,500 (90.75)	92.53 (0.91–0.93)
Yes	3,719 (9.25)	7.47 (0.07–0.08)
**Child given carbohydrate foods**		
No	32,448 (95.67)	95.95 (0.95–0.96)
Yes	1,469 (4.33)	4.04 (0.04–0.05)
**Child given protein foods**		
No	23,669 (69.66)	69.50 (0.68–0.71)
Yes	10,307 (30.34)	30.49 (0.29–0.32)
**Child given fatty foods**		
No	3,808 (44.19)	43.93 (0.42–0.46)
Yes	4,809 (55.81)	56.49 (0.54–0.58)
**Child given fruits**		
No	31,008 (91.29)	91.12 (0.90–0.92)
Yes	2,957 (8.71)	8.88 (0.08–0.10)
**Mothers' variables**
**Maternal age at first birth**		
11–19	13,246 (31.12)	31.54 (0.30–0.32)
20–28	26,916 (63.23)	63.13 (0.62–0.64)
29+	2,406 (5.65)	5.32 (0.05–0.06)
**Mother's education level**		
No education	8,440 (19.83)	19.87 (0.19–0.21)
Primary	3,804 (8.94)	9.01 (0.08–0.10)
Secondary	23,811 (55.94)	55.93 (0.55–0.57)
Higher	6,513 (15.30)	15.19 (0.14–0.16)
**Wealth index**		
Poor	16,637 (39.08)	38.30 (0.37–0.40)
Middle	8,844 (20.78)	24.00 (0.23–0.25)
Rich	17,087 (40.14)	37.69 (0.36–0.39)
**Mother's BMI**		
Not overweight/obese	28,637 (67.79)	65.08 (0.64–0.66)
Overweight/obese	13,605 (32.21)	34.92 (0.34–0.36)
**Mother's marital status**		
Not married	684 (1.61)	1.60 (0.01–0.02)
Married/cohabiting	41,884 (98.39)	98.40 (0.98–0.99)
**Antenatal visit**		
No	6,115 (14.37)	13.98 (0.13–0.15)
Yes	36,435 (85.63)	86.01 (0.85–0.87)
**Postnatal visit**		
No	22,673 (69.05)	67.78 (0.67–0.69)
Yes	10,164 (30.95)	32.21 (0.31–0.33)
**Fathers' variables**		
**Father's education level**		
No education	5,723 (13.45)	13.67 (0.13–0.14)
Primary	5,836 (13.71)	14.00 (0.13–0.15)
Secondary	23,940 (56.25)	55.58 (0.54–0.57)
Higher	7,060 (16.59)	16.74 (0.16–0.18)
**Current work status**		
Not working	37,240 (87.58)	87.55 (0.87–0.88)
Working	5,275 (12.41)	12.44 (0.12–0.13)

CI, Confidence interval; N, Total number of respondents.

For mothers' sociodemographic characteristics, more than a quarter (32%) were adolescents (11–19 years), the majority (63%) were young adults (20–28 years), and the remaining 5% were considered adults (29 years and above). Most mothers (56%) had attained secondary level education, one-fifth (20%) had no formal education, 15% had higher education, and the least (9%) had primary education. For wealth index, there was a similar proportions between poor and rich mothers, with each having ~38% and the remaining 24% being among those in the middle class in terms of wealth. Mothers who were overweight or obese were 34% whereas 65% were neither overweight nor obese. The vast majority (98%) were married and most of them (68%) did not visit a postnatal clinic after birth, whereas 32% attended postnatal clinics after birth. In terms of a child's father's educational level, more than half (56%) had secondary level education, followed by higher education (17%), primary education (14%), and no formal education (13%). Most (88%) of the fathers were not working at the time of the survey whereas 12% were gainfully employed (Table 2).

### Prevalence of overweight and obesity in Egypt

**Figure 1:**
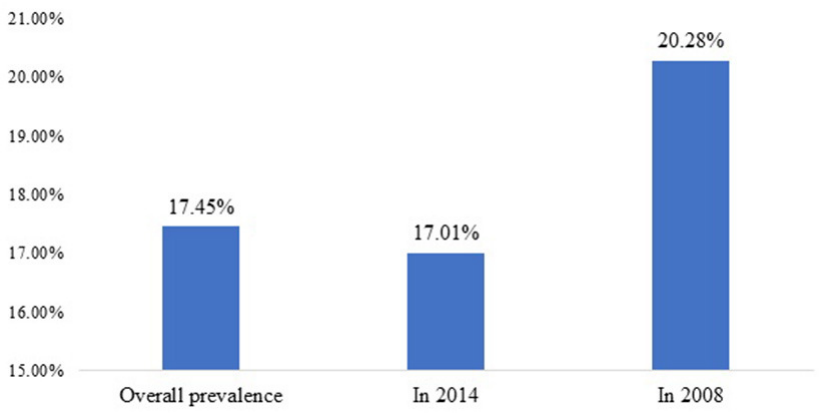
Prevalence of coexistence of overweight and obesity in Egypt, 2008 and 2014.

As shown in Figure 1, the overall prevalence of childhood overweight and obesity was ~17% (CI = 0.17–0.19). In 2014 alone, the prevalence rate was 16.9% (CI = 0.16–0.18), and in 2008 the prevalence was 20.3% (CI = 0.20–0.23). Although the prevalence of overnutrition is still high as of 2014, the findings show a slight decrease in childhood overweight and obesity between the two survey periods.

### Factors associated with overweight and obesity among under-five children in Egypt

There was a significant association between childhood overweight or obesity and the following factors: children aged 19 to 37 months (OR = 1.39, CI = 1.25–1.55, *p* = 0.001), children in rural residence (OR = 0.83, CI = 0.73–0.94, *p* = 0.003), children given protein foods (OR = 1.22, CI = 1.10–1.36, *p* = 0.001), children given fatty foods (OR = 1.45, CI = 1.26–1.67, *p* = 0.001), children given fruits (OR = 1.27, CI = 1.09–1.49, *p* = 0.001), children belonging to mothers aged 29 years or more (OR = 1.22, CI = 1.01–1.49, *p* = 0.04), children belonging to rich mothers (OR = 1.29, CI = 1.14–1.46, *p* = 0.001), children belonging to mothers who attended postnatal healthcare (OR = 1.12, CI = 1.02–1.24, *p* = 0.020), and children belonging to fathers who were gainfully employed (OR = 1.21, CI = 1.07–1.37, *p* = 0.002; Table 3).

**Table 3:** Logistic regression of associations between explanatory variables and overnutrition.

**Variable**	**Unadjusted**		**Adjusted**	
	**OR (95% CI)**	***p*-value**	**OR (95% CI)**	***p*-value**
**Child variables**				
**Age of child (months)**				
0–18 months	**Ref**.		**Ref**.	
19–37 months	1.39 (1.25–1.55)	***0.000***	1.32 (1.05–1.65)	***0.015***
38+ months	1.01 (0.89–1.14)	*0.904*	0.99 (0.74–1.31)	*0.925*
**Sex of child**				
Male	**Ref**.		**Ref**.	
Female	0.96 (0.88–1.04)	*0.364*	1.04 (0.85–1.28)	*0.705*
**Birth weight**				
< 2.5 kg	**Ref**.		**Ref**.	
2.5–4.0 kg	0.99 (0.83–1.18)	*0.951*	1.19 (0.82–1.71)	*0.360*
4.1 + kg	1.20 (0.8–1.75)	*0.336*	2.45 (1.25–4.81)	***0.010***
**Place of residence**				
Urban	**Ref**.		**Ref**.	
Rural	0.83 (0.73–0.94)	***0.003***	0.97 (0.75–1.25)	*0.801*
**Access to bicycle**				
No	**Ref**.		**Ref**.	
Yes	0.86 (0.72–1.03)	*0.103*	0.88 (0.59–1.30)	*0.514*
**Access to car**				
No	**Ref**.		**Ref**.	
Yes	0.99 (0.84–1.17)	*0.932*	1.23 (0.97–1.58)	*0.087*
**Child given carbohydrate foods**				
No	**Ref**.		**Ref**.	
Yes	0.96 (0.76–1.21)	*0.753*	0.92 (0.72–1.18)	*0.601*
**Child given protein foods (eggs and meats)**				
No	**Ref**.		**Ref**.	
Yes	1.22 (1.10–1.36)	***0.000***	1.29 (1.02–1.63)	***0.029***
**Child given fatty foods**				
No	**Ref**.		**Ref**.	
Yes	1.45 (1.26–1.67)	***0.000***	0.92 (0.73–1.17)	*0.524*
**Child given fruits**				
No	**Ref**.		**Ref**.	
Yes	1.27 (1.09–1.49)	***0.003***	1.22 (0.88–1.69)	*0.23*
**Mothers' variables**
**Maternal age at first birth**				
11–19	**Ref**.		**Ref**.	
20–28	1.01 (0.91–1.13)	*0.80*	1.15 (0.89–1.49)	*0.28*
29+	1.22 (1.01–1.49)	***0.04***	1.53 (0.92–2.54)	*0.10*
**Mother's education level**				
No education	**Ref**.		**Ref**.	
Primary	1.22 (1.03–1.46)	***0.021***	1.42 (0.87–2.31)	*0.16*
Secondary	1.24 (1.10–1.39)	***0.000***	1.31 (0.88–1.93)	*0.18*
Higher	1.33 (1.14–1.57)	***0.000***	1.14 (0.66–2.00)	*0.63*
**Wealth index**				
Poor	**Ref**.		**Ref**.	
Middle	1.02 (0.89–1.16)	*0.810*	2.02 (1.43–2.85)	***0.000***
Rich	1.29 (1.14–1.46)	***0.000***	2.19 (1.54–3.11)	***0.000***
**Mother's BMI**				
Not overweight/obese	**Ref**.		**Ref**.	
Overweight/obese	1.03 (0.94–1.15)	*0.464*	0.98 (0.75–1.26)	*0.851*
**Marital status**				
Not married	**Ref**.		**Ref**.	
Married	1.12 (0.74–1.67)	*0.593*	1.50 (0.52–4.33)	*0.448*
**Antenatal visit**				
No	**Ref**.		**Ref**.	
Yes	1.03 (0.91–1.16)	*0.609*	1.24 (0.91–1.69)	*0.175*
**Postnatal visit**				
No	**Ref**.		**Ref**.	
Yes	1.12 (1.02–1.24)	***0.020***	0.87 (0.67–1.12)	*0.052*
**Fathers' variables**				
**Father's education level**				
No education	**Ref**.		**Ref**.	
Primary	1.18 (0.99–1.42)	*0.478*	1.19 (0.74–1.92)	*0.474*
Secondary	1.05 (0.91–1.21)	*0.481*	1.13 (0.76–1.79)	*0.482*
Higher	1.23 (1.03–1.46)	*0.479*	1.06 (0.63–1.80)	*0.813*
**Current work status**				
Not working	**Ref**.		**Ref**.	
Working	1.21 (1.07–1.37)	***0.002***	1.08 (0.78–1.50)	*0.630*

CI, Confidence interval; Ref, reference; OR, Odds Ratio.

p-value < 0.05 considered statistically significant.

Italiced and bold values are significant p-values.

The model was then adjusted to confirm the associations between the identified explanatory variables and the childhood undernutrition indicators (overweight and obesity). We found that overweight and obesity were significantly associated with children aged 19 to 37 months **(**AOR = 1.32, CI = 1.05–1.65, *p* = 0.015), children with a birthweight of 4.1 kg and above (AOR = 2.45, CI = 1.25–4.81*, p* = 0.010), children given protein foods (AOR = 1.29, CI = 1.02–1.63, *p* = 0.029), and rich mothers (AOR = 2.19, CI = 1.54–3.11, *p* = 0.001; Table 3).

These results imply that children aged 19–37 months had 1.32 higher odds of becoming overweight or obese compared to those younger and older (<19 and >37 months). Those who weighed more than 4.1 kg had 2.45 times higher odds of being overweight or obese compared to those who weighed lesser. Children who significantly consumed protein foods had 1.29 higher odds of becoming overweight or obese relative to those who consumed other food types. With regards to maternal factors, those belonging to rich mothers had 2.19 higher odds of becoming overweight or obese compared to those belonging to poor mothers and mothers with an average level of wealth (Table 3).

## Discussion

This study aimed to examine the prevalence and correlates of overweight and obesity among children under 5 years in Egypt. The findings revealed that the overall prevalence of overweight and obesity was approximately 17% which corresponds with previous community-based studies in Egypt which found an overnutrition prevalence rate ranging from 15 to 18% (3, 22). Our reported prevalence rate for Egypt is nearly twice the average rate for the world, which is estimated at 8.6%, indicating a serious childhood overnutrition problem in Egypt and the need for prompt actions to reduce this significant health burden (8).

The study also found several socio-demographic factors significantly associated with overweight and obesity among children under 5 years in Egypt. Notably, the childhood overnutrition indicators were significantly associated with children aged between 19 and 37 months, children with birthweights of 4.1 kg and above, children given large amounts of protein foods, and children belonging to rich mothers. The findings that children within the age bracket of 19–37 months had higher odds of developing childhood overweight or obesity is quite difficult to explain as there is a lack of literature on this topic (23). However, limited evidence shows that toddlers around that age bracket often engage in emotional eating behavior and often use food as a form of compensation or a soothing intervention when they feel upset which may put them at serious risk of being overweight or obese (24). Additionally, parenting styles and feeding practices may contribute to overweight or obesity among such infants (25–28). A systematic review has noted strong associations between indulgent or uninvolved parenting style and high childhood BMI whereas an authoritative parenting style was reported to be associated with a healthy BMI (28). Indulgent parenting style means parenting styles based on a combination of low demandingness, high responsiveness to a child's needs, and few rules; indulgent parenting style implies parenting styles associated with both low demandingness and low responsiveness; and authoritative parenting styles are parenting styles associated with a high level of demandingness, numerous rules, and high responsiveness to the needs of a child (28). Regarding feeding practices, the study further emphasized that authoritative feeding styles are associated with healthy BMIs whereas indulgent feeding styles are associated with a higher risk of overweight or obesity (28). Nevertheless, the systematic review and other available literature on parenting feeding practices were not decomposed according to age categories, and therefore, an in-depth exploration of the interplay of childhood age and parental feeding practices with overweight or obesity will be timely and important (25–28).

Consistent with previous studies (29, 30), we found that children who weighed 4.1 kg or higher at birth were more likely to experience childhood overweight or obesity. In line with our findings, a study in Sweden has indicated that males and females with birth weights above three standard deviation scores had higher risks of obesity, ranging from 2.46 to 1.85, respectively (30). In contrast, biological evidence shows that children with low birth weight have a higher concentration of plasma leptin which increases their obesity risks (31). Several previous studies have also indicated that low birth weights are associated with higher BMI *z*-scores and higher odds of obesity (32, 33). Casey et al. (33) for instance, reported an increase in obesity in a cohort of low-birth-weight preterm infants and reported a proportional increase in obesity from 2.3% at the age of 3 years to 6.1% prevalence by the age of 5 years. Together, these findings demonstrate an interesting paradox in terms of associations between birth weights and risks of overweight or obesity which may be attributed to differences in samples and confounding variables, such as variations in residence. To adequately understand the impact of birth weights on risks of overweight or obesity, it will be important to employ a longitudinal approach whereby the same cohort of infants will be studied over time. Nevertheless, our study contributes to the growing evidence on associations between high birth weights and risks of being overweight or obese in subsequent years of a child's life.

With regards to food consumption, children who consumed protein foods such as meat and eggs had a greater risk of overweight and obesity. This finding is in line with several previous studies (3, 34). A plausible reason for this outcome is that a high intake of protein foods is likely to lead to excess storage of body fats which eventually leads to a higher risk of overweight or obesity (24, 26). It needs to be however acknowledged that the assessment of food intake in the DHS data were limited to “yes” and “no” responses, hence this finding should be taken with caution. Consistent with previous studies (27, 28, 35), we also found that children belonging to wealthy mothers had a higher risk of overweight and obesity. This is probably because children in rich households may be exposed to high-calorie foods and sedentary lifestyles which puts them at risk of overweight and obesity (36, 37).

While taking due caution in making policy suggestions based on findings from this cross-sectional data, our findings which are based on over 40,000 under-five children provide a comprehensive and important “wake-up” call to policymakers and health advocates about the need to pay close attention to childhood overnutrition in Egypt. Particularly, interventions aiming to reduce childhood undernutrition need to be cautious of the possibilities of causing overnutrition after the implementation of food intake interventions. Additionally, our findings indicate that the growing overnutrition problem in Egypt (North Africa) can be effectively reduced if attention is paid to modifiable risk factors, such as sedentariness and overfeeding of toddlers with high protein foods, especially, by wealthy mothers (12). Supportive interventions are important in shaping people's choices, especially, in choosing healthier foods for their children and engaging in regular physical activity (12). At the individual level, parents can limit the provision of foods high in proteins to toddlers and increase the consumption of fruit and vegetables (38, 39). We believe that these factors are critical in promoting optimal health for children aged under 5 years in Egypt and even beyond.

Recent studies on overweight and obesity in Africa have indicated a potential double burden of undernutrition and overnutrition in the continent (40, 41). However, the previous studies have been largely focused on adult populations in Sub-Saharan Africa with limited studies focusing on children in North Africa, although anecdotal evidence shows that many North African countries have a potentially high prevalence of childhood overnutrition. Using Egypt as a case study, we have successfully reported the prevalence of overweight and obesity among children under-5 years and the risk factors associated with these overnutrition indicators. The potential burden of childhood overnutrition in Africa has received much attention in recent years. Modifiable risk factors may be important contributors to overnutrition, and this study is one of the early research projects examining this emerging problem for early interventions. Findings from this study provide useful calls for researchers, health promoters, and policymakers to consider seriously the emerging problem of childhood overnutrition in Egypt and North Africa.

### Limitations of the study

By examining the prevalence and factors associated with childhood overnutrition in Egypt, this study provides a timely empirical contribution to the literature examining whether childhood overnutrition is indeed a growing problem in North Africa.

Although this study makes a noteworthy contribution to the literature on childhood overnutrition including the pooling of large samples of children under-5 years from two standard EDHS, and therefore increasing the statistical power, some limitations can be observed. First, the data is limited to ever-married women with children under 5 years which may limit the generalizability of the findings. The study is cross-sectional and therefore we were unable to make causal inferences. Also, some variables were self-reported which may be subject to recall and social desirability biases. There was evidence of missing responses for some variables which made data cleaning and validation complicated. Also, almost all the variables were used in the regression analysis, and this is likely to contribute to over-adjustment bias. However, all the variables were important possible risk factors for childhood overweight or obesity in Egypt. Although we used the latest EDHS data, the available data were gathered in 2008 and 2014 which are quite old and may lack some currency. Therefore, the prevalence rates should be taken cautiously. Lastly, food consumption frequency, timing, and portions were not captured in the data (i.e., the timing, portions, and frequency of protein intake, fat intake, fruit intake, or carbohydrate intake were not captured, and the questions were asked based on self-observational measures). Responses to dietary intake were limited to “yes” or “no” answers, making it hard to examine less healthy dietary intake. While this might have impacted the significance of the findings on food intake, the current findings provide an important indication of the need to pay close attention to food behavior among under-five children in Egypt.

## Conclusion

We conclude that overnutrition (overweight and obesity) is highly prevalent among children under 5 years in Egypt. Approximately one in every six children (17%) included in our sample were either overweight or obese. We also found that a child's age (19–37 months), birth weight (>4 kg), protein consumption levels, and mothers' wealth status were significantly associated with the likelihood of overweight and obesity among children under-5 years in Egypt. This is a significant public health concern as overweight or obesity increases risk of numerous health issues, such as cardiovascular diseases, musculoskeletal diseases, diabetes, stroke, and some cancers (12). These findings adequately address the objectives of this study and indicate the need for early and urgent strategies to mitigate childhood overnutrition in Egypt.

## Data availability statement

The datasets presented in this study can be found in online repositories. The names of the repository/repositories and accession number(s) can be found at: http://dhsprogram.com/data/available-datasets.cf.

## Ethics statement

Ethical approval was not sought for this study since our analysis was based on publicly available data. However, the DHS reports that both written and verbal informed consent were obtained from all participants. Before the commencement of the survey, ethical clearance was sought and all ethical guidelines governing the use of human subjects were strictly adhered to and methods were carried out in accordance with the relevant guidelines and regulations by the Declaration of Helsinki.

## Author contributions

IA conceived the study, reviewed the analyses, and supervised the entire study. EO conducted the multilevel analysis. EO and IA drafted and critically reviewed the manuscript. All authors read and approved the final manuscript.
